# Akabane virus isolated from biting midges and its infection in local domestic animal, Yunnan, China: a field and laboratory investigation

**DOI:** 10.3389/fcimb.2024.1434045

**Published:** 2025-01-17

**Authors:** Jinxin Meng, Yuwen He, Nan Li, Zhenxing Yang, Si Fu, Dongmei Wang, Aiguo Xin, Jinglin Wang, Guodong Liang

**Affiliations:** ^1^ Yunnan Tropical and Subtropical Animal Viral Disease Laboratory, Yunnan Animal Science and Veterinary Institute, Kunming, Yunnan, China; ^2^ Jiangcheng County Animal Disease Prevention and Control Center, Jiangcheng, Yunnan, China; ^3^ National Institute for Viral Disease Control and Prevention, Chinese Center for Disease Control and Prevention, Beijing, China

**Keywords:** Akabane virus, biting midges, domestic animal, virus isolation, neutralizing antibody

## Abstract

**Introduction:**

We verified that Akabane virus (AKAV) is transmitted through biting midges and infects local domestic animals.

**Methods:**

In 2013, viruses were isolate from biting midges in Yunnan, China, using BHK-21 and C6/36 cells. Two AKAV strains (No. 52 and 55) that induced cytopathogenic effects (CPE) in BHK-21, MDBK, and Vero cells were characterized.

**Results:**

The complete genomic sequence of both viruses consisted three RNA segments (S, M, and L). The S segment (856 nucleotides) encoded a 233-amino-acid nucleocapsid protein and a 91-amino-acid nonstructural protein, while the M segment (4309 nucleotides) encoded a 1401-amino-acid polyprotein. The L segment (6869 nucleotides) encoded a 2511-amino-acid RNA-dependent RNA polymerase. Phylogenetic analysis revealed that specimen Nos. 52 and 55 clustered with AKAV genotype Ia viruses isolated from Asia. The AKAV strain (55) neutralizing antibody exhibited a total positive rate of 43.55% (202/466) against serum samples from cattle and goats collected in Yunnan Province. Specifically, the positive rates were 48.77% (139/285) for cattle and 34.81% (63/181) for goats. Neutralizing antibody titers in cattle (1:32–1:128) were higher than those in goats (1:4–1:16).

**Discussion:**

This study represents the first isolation of AKAV from biting midges in China, along with the detection of high neutralizing antibody titers against AKAV in the serum samples of local cattle and goats. These findings suggested that biting midges are involved in AKAV transmission among domestic animals in Yunnan Province, China.

## 1 Introduction

Akabane virus (AKAV) is a culicoides-borne arbovirus that primarily affects cattle and small ruminants (Tohru et al., 2019). AKAV infection can result in congenital defects, abortion, premature birth, stillbirth (Kirkland, 2015), and even encephalitis in pregnant animals (Lee et al., 2002). Historical outbreaks of AKAV-induced animal diseases have caused significant economic losses. For instance, in 2010, AKAV led to encephalomyelitis in over 500 cattle in South Korea (Jeong et al., 2017), and in 2011, an outbreak of AKAV-induced encephalomyelitis occurred Shimane Prefecture, western Japan (Hayama et al., 2016). Between 2013 and 2016, numerous adult and juvenile bamboo rats in Guangxi Province, China, succumbed to AKAV infection and died (Tang et al., 2017). The spread of AKAV has consequently imposed substantial losses and financial burdens on the animal husbandry.

AKAV is crucial vectors by biting midges (Kurogi et al., 1987; Jeong et al., 2017), and its spread is largely influenced by the geographical distribution of these vectors. The virus has been detected in various parts of the temperate and subtropical regions, including Australia (Della-Porta et al., 1977), Asia (Bryant et al., 2005; Hayama et al., 2016; Jeong et al., 2017), the Middle East (Stram et al., 2004), and parts of African (Metselaar and Robin, 1976). AKAV was first isolated from mosquito specimens in Japan (Oya et al., 1961). Recently, the virus was detected in Yunnan Province, China, and has been isolated from local *Anopheles vagas*, *Culex quinquefasciatus* (Feng et al., 2015), as well as from sentinel goats, and cattle (Meng et al., 2019, 2020; Gao et al., 2022). Yunnan’s subtropical climate, diverse ecosystems, and extensive agricultural activities provide optimal conditions for the breeding of biting midges, the primary vector of AKAV. Moreover, Yunnan’s borders with several Southeast Asian countries, where cross-border livestock movement is common, further heightens the risk of AKAV introduction and spread. However, despite the crucial role of biting midges in AKAV transmission, the virus has not yet been isolated from biting midges collected on the Chinese mainland.

In this study, two AKAV strains (No. 52 and 55) were isolated from biting midges collected near cattle shelters in Shizong County, Yunnan Province, China, in 2013. The complete genome of both strains was sequenced, and neutralizing antibodies to AKAV were detected in domestic animals. These findings suggest that AKAV is present in natural populations of biting midges in mainland China. Furthermore, this virus transmitted by these biting midges appears to have established a natural cycle involving local domestic animals.

## 2 Materials and methods

### 2.1 Collection of biting midge samples

Biting midges were collected using light traps (Gongfu Xiaoshua, 12 V, 300 mA, Wuhan, China) placed near cattle, goats, and pig helters in Wulong Township, Shizong County, Yunnan Province, in July 2013. The collection occurred overnight, from 18:00 to 8:00 the following morning (Zhai et al., 2006).

The collected biting midges were killed by freezing at −20°C for 30 minutes and identified based on their morphological characteristics. They were then pooled into groups, with each group containing approximately 50 biting midges, and placed into individua tubes. Each tube was labeled and stored in liquid nitrogen until transported to the laboratory for virus isolation.

### 2.2 Collection of animal serum samples

Cattle and goats over one year of age, with no record of AKAV vaccination, were randomly selected from Shizong, Jiangcheng, and Shuangjiang counties in Yunnan Province. Jugular vein blood samples (5 mL) were collected from each animal using vacuum blood collection tubes. The blood was allowed to coagulate, after which the tubes were centrifuged at 3000 rpm for 10 minutes. The separated serum samples were then frozen at −20°C until further use.

### 2.3 Cells culture and isolation of viruses

Baby hamster kidney (BHK-21)cells, Madin-Darby bovine kidney (MDBK) cells, African green monkey kidney (VERO) cells, and *Aedes albopictus* cells (C6/36) were stored in the Yunnan tropical subtropical animal virus diseases laboratory. BHK-21, MDBK, and VERO were cultured in Dulbecco’s modified Eagle’s medium (DMEM) at 37°C, while C6/36 was cultured in Roswell Park Memorial Institute medium 1640 at 28°C. All cells were grown in respective media, supplemented with 10% fetal calf serum, 100 U/mL penicillin, and 100 μg/mL streptomycin, at a pH of 7.4.

Biting midge pools were removed from liquid nitrogen and homogenized in 1 mL of grinding fluid in sterile Eppendorf tube using a high-throughput tissue grinder (QIAGEN TissueLyser II, Germany) at −20°C for 10 minutes at a frequency of 30 times per second. The tubes were then centrifuged at 8000 rpm for 10 minutes, and 100 μL of supernatants was inoculated into each well containing a monolayer of C6/36, BHK-21, MDBK, or VERO cells in 24-well plates respectively. The cells were observed daily (days 1–7 post-inoculation) for cytopathic effects (CPE). A blind passage was sustained for three generations, and the supernatants from CPE-positive cultures were used for expanded cultivation and stored at –80°C for identification.

### 2.4 Preliminary identification of virus

Viral RNA was extracted from the CPE-positive cell culture supernatants using RNAiso Plus (TaKaRa, Dalian, China), following the manufacturer’s instructions.

The virus was identified through reverse transcriptase-polymerase chain reaction (RT-PCR). First-strand cDNA was synthesized using random primers P(d)N6 (TaKaRa, Dalian, China) and PrimeScript1 Reverse Transcriptase (TaKaRa, Dalian, China), as per the manufacturer’s instructions. The cDNA was then amplified via PCR using specific primers for Flavivirus (Kuno, 1998), Alphavirus (Pfeffer et al., 1997), and Simbu serogroup (Camarão et al., 2019). The PCR products were separated by electrophoresis on 1.3% agarose gel containing nucleic acid dye 1× TAE buffer. The results were visualized under ultraviolet light and sequenced using next-generation sequencing.

### 2.5 Full genome sequencing

The PCR primers were designed based on the AKAV strain sequence DHL10M110 (KY284023, KY284022, KY284021) in GenBank (**Table 1**). RT-PCR was performed on viral RNA to obtain all AKAV gene fragments. The amplified DNA fragments were purified using a DNA Fragment Purification Kit (TaKaRa, Dalian, China). Then cloned into the pMD19-T cloning vector (TakaRa, Dalian, China) and transformed into the chemically competent *Escherichia coli* DH5α cells (TakaRa, Dalian, China). The PCR products and the selected transformed colonies were cultured and sequenced by Beijing Tsingke Biotech (Beijing, China).

**Table 1:** The primers used for the amplification of the S, M, L segments of the Akabane virus genome.

Gene	Primer	Sequence 5’→3’	Product size (bp)	°C
S	AKS1F	AGTAGTGAACTCCACTATTAAC	856	46
	AKS856R	AGTAGTGTGCTCCACTAATT		
M	M1F	AGTAGTGAACTACCACAACAAAATG	1359	55
	M1359R	TACTTGGCGGTTTTGTATTTC		
	M1262F	TTCACATCTCATTGCCTTAC	1632	51
	M2893R	GAAACCCTGCGATAGTCC		
	M2597F	GATTGCTACCCTTATTATTC	1659	46
	M4255R	CCTCCCGTTATGTCTATT		
L	L1F	TAGTGTACCCCTAAATACAAC	510	47
	L510R	TTGTAAAACCAAGTAAAATCT		
	L201F	TCCTCCTGGATTTCTTAT	1483	47
	L1683R	GTTGCCCTCTTTGTCTTA		
	L1566F	ACATGCTGGCAGTGTCGC	1239	55
	L2804R	CGGCTGGTCTGTCTGTTATTT		
	L2627F	TACACAATAGCAAACCCAGA	931	50
	L3557R	TTGAAGTGTGGTTATCATCTG		
	L3408F	GGCTTCAGGGCAATCTAA	815	53
	L4222R	GCCATCGTCGCTACTCAT		
	L3786F	ATGAGGATACAGCGAGCAG	1644	52
	L5429R	TTGTCCTAAGTCGGGAGG		
	L4929F	ACCATCTGGAGGAGTTCA	927	48
	L5855R	TTGTTTGGCTCAATCTGT		
	L5447F	TTTTACATGGGCGATTTG	1277	51
	L6723R	GCTTGCGTACTCGTCTTT		
	L5845F	GAGCCAAACAAGGTCAAG	1015	50
	L6859R	GCCCCTAAATGCAATAAT		

The initial sequence assembly and analyses were performed using Seqman software in the DNAStar software package. The open reading frame (ORF) sequences of the S, M, and L segments of AKAV strains isolated from China, Japan, South Korea, Australia, and Kenya, as well as Aino virus strains belonging to the Simbu serogroup, were downloaded from GenBank. Homology and alignment analyses were conducted using MegAlign software in the DNAStar software package. With MEGA 6.1 based on the maximum likelihood assay, phylogenetic and evolutionary analyses were conducted. The bootstrap value was 1,000.

### 2.6 Viral neutralization antibody

Antibodies in serum samples were detected using a neutralization test. After heat treatment at 56°C for 30 minutes, each serum sample was diluted to 1:4, 1:8, 1:16, 1:32, 1:64, 1:128, 1:256, and 1:512, with two replicates per dilution. Each serum dilution (50 μL) was mixed with an equal volume of the virus supernatant (100 TCID_50_/50 μL) and inoculated into 96-well plates. Viral supernatants of 100TCID_50_, 10TCID_50_, and 1TCID_50_, along with a blank control, were included as the controls. The plates were shaken for 5 min and then incubated for 1 hour at 37°C under 5% CO_2_ to allow for neutralization. Following this, 100 μL of BHK-21 cell suspension (1 × 10^6^ cells/mL) was added to each well. The plates were observed daily for 7 days, and the experimental results were evaluated according to previously reported criteria (Meng et al., 2023). A serum sample was considered positive for virus neutralizing antibody if the titer was ≥1:4 (Xu et al., 2007).

## 3 Results

### 3.1 Sample collection statistics

In July 2013, a total of 3704 biting midges were collected from Shizong County, comprising 2437 (49 pools), 117 (2 pools), and 1150 (23 pools) from cattle, goat, and pig helters, respectively. In total, 74 pools of biting midges were collected.

Additionally, 466 serum samples were collected from three sampling sites, snamely 147 (cattle 98 and goats 49) from Shizong County, 154 (cattle 101 and goats 53) from Jiangcheng County, and 165 (cattle 86 and goats 79) from Shuangjiang County.

### 3.2 Virus isolation and identification

**Figure 1:**
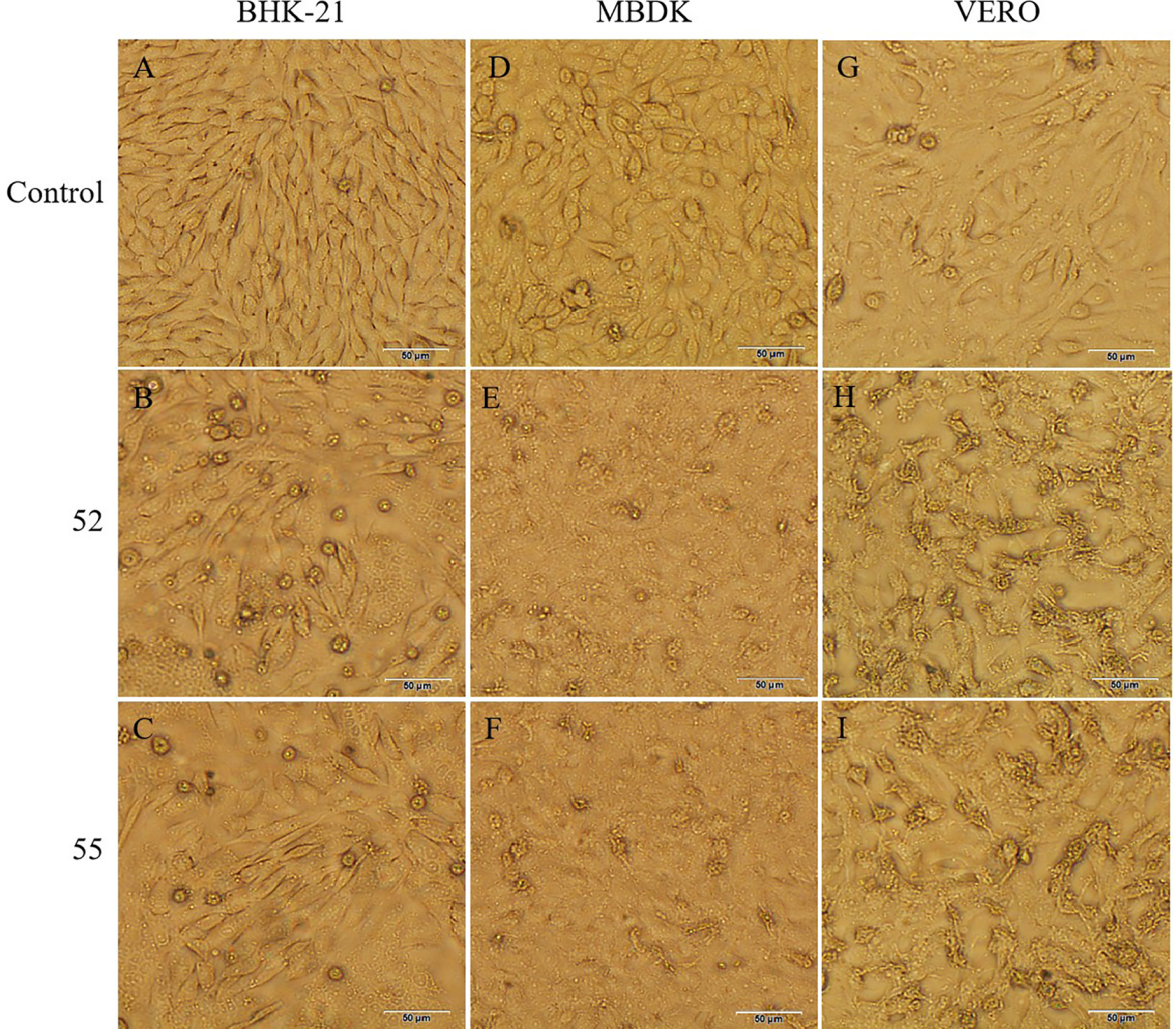
CPE caused by 52 and 55 on BHK-21, MDBK, and VERO cells 48 h postinoculation (100×). **(A)** Control BHK-21 cells. **(B)** Cytopathic effect of 52 in BHK-21 cells. **(C)** Cytopathic effect of 55 in BHK-21 cells. **(D)** Control MDBK cells. **(E)** Cytopathic effect of 52 in MDBK cells. **(F)** Cytopathic effect of 55 in MDBK cells. **(G)** Control VERO cells. **(H)** Cytopathic effect of 52 in VERO cells. **(I)** Cytopathic effect of 55 in VERO cells. Scale bar 50μm.

Two pools of biting midges (No. 52, 55) from cattle helters induced CPE in the BHK-21, MDBK, and VERO cells (**Figure 1**). The BHK-21 cells became spherical and detached after 24 hours, with CPE reaching 50% at 48 hours post-inoculation. The MDBK cells fragmentized and detached after 24 hours, with CPE reaching 50% at 48 hours post-inoculation. The VERO cells exhibited reticulated and detached after 24 hours, with CPE reaching 50% at 48 hours. No significant CPE was observed in the C6/36 cells.

Positive samples for strains 52 and 55 were amplified using RT-PCR with Simbu serogroup primers, yielding approximately 600 nucleotides for sequencing. BLAST analyzed on NCBI revealed that the obtained sequences had over 97% similarity to the S segments of AKAV, indicating that viruses 52 and 55 are closely related to AKAV.

### 3.3 Genome organization and characteristics of AKAV

The S, M, and L segments of the two virus isolates were obtained through whole genome sequencing. The S segment was 856-nucleotide long and contained two overlapping open reading frames (ORFs) of 699 and 285 nucleotides, respectively, encoding a 233-amino-acid nucleocapsid (N) protein and a 91-amino-acid nonstructural (NSs) protein, respectively. The M segment contained a 4,206-nucleotide ORF encoding 1,401 amino acids, while the L segment contained a 6756-nucleotide ORF encoding 2251 amino acids. The complete AKAV genome sequences for strains 52 and 55 were submitted to GenBank (**Table 2**).

**Table 2:** Length and number of nucleotide sequences of the complete genome of AKAV strains newly isolated from Yunnan.

Segment	Strain	Accession	Length	GC content	5’UTR	ORF	3’UTR
S	52	OR387104	856 nt	47.15	1-33 (33 nt)	N 34-735 (702 nt, 233aa) NSs 59-334 (276 nt, 91aa)	736-856 (121 nt)
	55	OR387105		47.15			
M	52	OR387106	4309 nt	37.47	1-22 (22 nt)	23-4228 (4206 nt, 1401aa)	4229-4309 (81 nt)
	55	OR387107		37.45			
L	52	OR387108	6869 nt	35.33	1-30 (30 nt)	31-6786 (6756 nt, 2251aa)	6787-6869 (83 nt)
	55	OR387109		35.32			

The nucleotide (and amino acids) of S, M, and L segments between the 52 and 55 strains were 100% (100% for N and NSs), 99.7% (99.6%), and 99.9% (99.9%), respectively. The nucleotide and amino acid homology of strain 55 was compared with 12 representative AKAV strains from China, Japan, South Korea, Australia, and Kenya (**Table 3**). The nucleotides (amino acids) of S, M, and L segments between strain 55 and genogroup I exhibited 95.6%–99.4% (98.3%–100% for N protein and 95.6%–97.8% for NSs protein), 91.9%–98.9% (95.4%–99.3%), and 94.8%–99.1%(98.6%–99.6%) identity, respectively. The nucleotides (amino acids) of S, M, and L segments between strain 55 and genogroup II exhibited 95.4%–96.4% (98.7% for N protein and 95.6%–97.8% for NSs protein), 87.5%–88.3% (93.6%–94.4%), and 92.0%–92.7% (97.6%–97.8%) identity, respectively. The nucleotides (amino acids) of S and M segments between strain 55 and genogroup III exhibited 92.6% (97.4% for N protein and 94.5% for NSs protein) and 84.3% (91.9%) identity, respectively. The nucleotides (amino acids) of S and M segments between strain 55 and genogroup IV displayed 83.8% (90.6% for N protein and 73.6% for NSs protein) and 70.5% (74.2%) identity, respectively. The 52 and 55 strains shared a high nucleotide and amino acid homology with genogroup Ia. Among them, the CX-01 strain isolated from Yunnan goats exhibited the highest homology.

**Table 3:** Nucleotide and amino acid sequence identity (%) between 52, 55, and representatives of recognized AKAV.

Genogroup	Strain	Source	Country	55(S)			55(M)		55(L)	
				nt	aa (N)	aa (NSs)	nt	aa	nt	aa
Ia	52	Midge	China	100	100	100	99.7	99.6	99.9	99.9
	CX-01	Goat	China	99.4	100	96.7	98.9	99.3	99.1	99.6
	DHL10M110	*Anopheles vagus*	China	97.4	99.6	97.8	91.9	95.4	97.8	99.5
	GXLCH02	Rhizomys pruinosus	China	97.7	99.1	97.8	97.4	98.3	94.8	98.7
	HN10174	*Culex quinquefasciatus*	China	97.9	99.1	97.8	91.9	96.2	95.5	98.9
	NM/BS/1	Bovine serum	China	98.1	99.1	97.8	91.9	96.1	_	_
	CY-77	Bovine erythrocyte	Chinese Taiwan	96.7	99.6	96.7	96.7	98.0	_	_
	AKAV-7/SKR/2010	Cattle brain	South Korea	95.6	98.7	95.6	98.1	98.6	95.3	98.6
Ib	FO-90-3	*Culicoides*	Japan	95.6	98.3	96.7	91.9	95.5	_	_
	KSB-2/C/90	*Culicoides*	Japan	95.6	98.3	96.7	91.9	95.4	_	_
II	OBE-1	Bovine aborted fetus	Japan	95.4	98.7	95.6	87.6	93.9	92.0	97.6
	JaGAr39	*Aedes vexans*	Japan	96.4	98.7	97.8	88.3	94.4	_	_
	TS-C2	Bovine fetus	Japan	95.6	98.7	95.6	87.5	93.6	92.0	97.6
	JaLAB39	*Aedes vexans*	Australia	96.4	98.7	97.8	88.3	94.4	92.7	97.8
III	B8935	*Culicoides brevitarsis*	Australia	92.6	97.4	94.5	84.3	91.9	_	_
IV	MP496	*Culicoides brevitarsis*	Kenya	83.8	90.6	73.6	70.5	74.2	_	_

Representative AKAV strains from various sources and countries were selected, encompassing four genogroups Ia, Ib, II, III, and IV. In total, 16 strains were analyzed for amino acid variation. Compared to the Chinese strain GXLCH02, strains 52 and 55 isolated from Yunnan biting midges, exhibited unique amino acid mutations in the NSs protein (position 79) and the L segment (position 763). The four strains isolated from Yunnan shared the same amino acid mutation at position 563 of the L segment. Additionally, eight strains from China displayed a common amino acid mutation at position 206 of the N protein, while seven strains from the Chinese mainland showed the same amino acid mutation at position 76 of the NSs protein. Genotype Ia strains contained unique amino acid variations at four positions (255, 610, 936, and 1279) in the M segments and five position (145, 1106, 1185, 1335 and 1524) in the L segments. Furthermore, genotype I strains exhibited three unique amino acid sites (positions 14, 758, and 1077) in the M segment (**Table 4**).

**Table 4:** Amino acid changes in the deduced sequences of the N, NSs, M, and L proteins of AKAV strains compared with those of 52 and 55.

	Strain	Country		S		M													L							
			N	NSs																						
Ia	GXLCH02	China	206	76	79	14	255	325	348	352	610	716	758	829	936	1077	1279	1295	145	288	563	763	1106	1185	1335	1524
			N	T	Q	I	L	S	A	A	H	I	T	I	S	G	S	G	N	S	H	A	Q	S	A	C
	52	China	**·**	**·**	R	**·**	**·**	G	E	T	**·**	T	**·**	T	**·**	**·**	**·**	D	**·**	N	Q	T	**·**	**·**	**·**	**·**
	55	China	**·**	**·**	R	**·**	**·**	G	E	T	**·**	T	**·**	T	**·**	**·**	**·**	D	**·**	N	Q	T	**·**	**·**	**·**	**·**
	CX-01	China	**·**	**·**	**·**	**·**	**·**	G	E	T	**·**	T	**·**	T	**·**	**·**	**·**	D	**·**	N	Q	**·**	**·**	**·**	**·**	**·**
	DHL10M110	China	**·**	**·**	**·**	**·**	**·**	**·**	**·**	**·**	**·**	**·**	**·**	**·**	**·**	**·**	**·**	**·**	**·**	N	Q	**·**	**·**	**·**	**·**	**·**
	HN10174	China	**·**	**·**	**·**	**·**	**·**	**·**	**·**	**·**	**·**	**·**	**·**	**·**	**·**	**·**	**·**	**·**	**·**	**·**	**·**	**·**	**·**	**·**	**·**	**·**
	NM/BS/1	China	**·**	**·**	**·**	**·**	**·**	**·**	**·**	**·**	**·**	**·**	**·**	**·**	**·**	**·**	**·**	**·**	—	—	—	—	—	—	—	—
	CY-77	Chinese Taiwan	**·**	I	**·**	**·**	**·**	**·**	**·**	**·**	**·**	**·**	**·**	**·**	**·**	**·**	**·**	**·**	—	—	—	—	—	—	—	—
	KM-2/Br/06	Japan	S	I	**·**	**·**	**·**	**·**	**·**	**·**	**·**	**·**	**·**	**·**	**·**	**·**	**·**	**·**	**·**	**·**	**·**	**·**	**·**	**·**	**·**	**·**
	AKAV-7/SKR/2010	South Korea	S	I	**·**	**·**	**·**	**·**	**·**	**·**	**·**	**·**	**·**	**·**	**·**	**·**	**·**	**·**	**·**	**·**	**·**	**·**	**·**	**·**	**·**	**·**
Ib	KSB-2/C/90	Japan	S	I	**·**	**·**	Q	**·**	**·**	**·**	Q	**·**	**·**	**·**	G	**·**	N	**·**	—	—	—	—	—	—	—	—
II	OBE-1	Japan	S	I	**·**	T	Q	**·**	**·**	**·**	Q	**·**	A	**·**	G	S	N	**·**	H	**·**	**·**	**·**	K	P	S	S
	JaLAB39	Australia	S	I	**·**	A	Q	**·**	**·**	**·**	Q	**·**	A	**·**	G	S	N	**·**	H	**·**	**·**	**·**	R	P	S	S
III	B8935	Australia	S	I	**·**	T	Q	**·**	T	**·**	Q	**·**	V	**·**	G	S	N	D	H	N	**·**	**·**	K	P	S	S
IV	MP496	Kenya	S	I	**·**	A	E	V	E	N	A	**·**	I	T	G	S	N	**·**	—	—	—	—	—	—	—	—

### 3.4 Phylogenetic classification of 52 and 55

**Figure 2:**
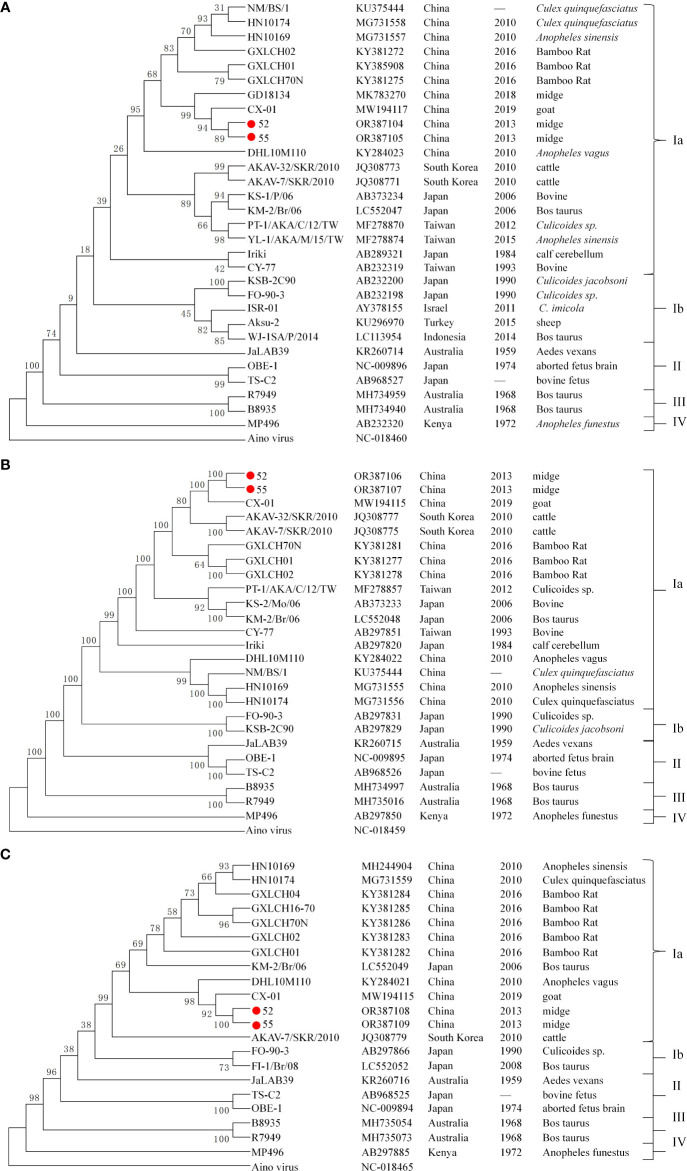
Phylogenetic trees of AKAV based on the ORF nucleotide sequence of S segment N protein **(A)**, M segment **(B)**, and L segment **(C)**. The trees were constructed using the maximum likelihood assay. Sequences obtained in this study are marked with a round red spot (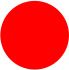). The nucleotide sequences of other AKAV strains used here were obtained from GenBank.

**Figure 2** illustrates the phylogenetic trees based on the nucleotide sequences of the S segment N protein (**Figure 2A**), M segment (**Figure 2B**), and L segment (**Figure 2C**) for viruses 52 and 55, alongside representative AKAV strain from various countries, hosts, and vectors, as well as the Aino virus strain from the Simbu serogroup. The analysis revealed that AKAV strains fall into genotypes I–IV, with genotype I further subdivided into two branches: Ia and Ib. Phylogenetic analysis of the N protein indicated that the viruses 52 and 55, isolated from biting midges in the study, belong to the branch as genotype Ia. Notably, AKAV isolates from the Chinese mainland formed a separate branch within the genotype Ia cluster, separate from viruses isolated from Chinese Taiwan, Japan, and South Korea. The phylogenetic tree based on the nucleotide sequences of the M and L segments further confirmed that strains 52 and 55 are classified within genotype Ia, with isolates from China, Japan, and Korea clustering closely in similar evolutionary groups.

### 3.5 Prevalence of AKAV antibodies in cattle and goats

In total, 466 serum samples were collected from Shizong, Jiangcheng, and Shuangjiang counties and tested for AKAV neutralizing antibodies. The neutralizing antibody titer for AKAV was ≥1:4 in 202 serum samples, resulting in a positive rate of 43.35% (200/466). The detection of seropositive cattle and goats in this study suggests natural exposure to AKAV.

**Figure 3:**
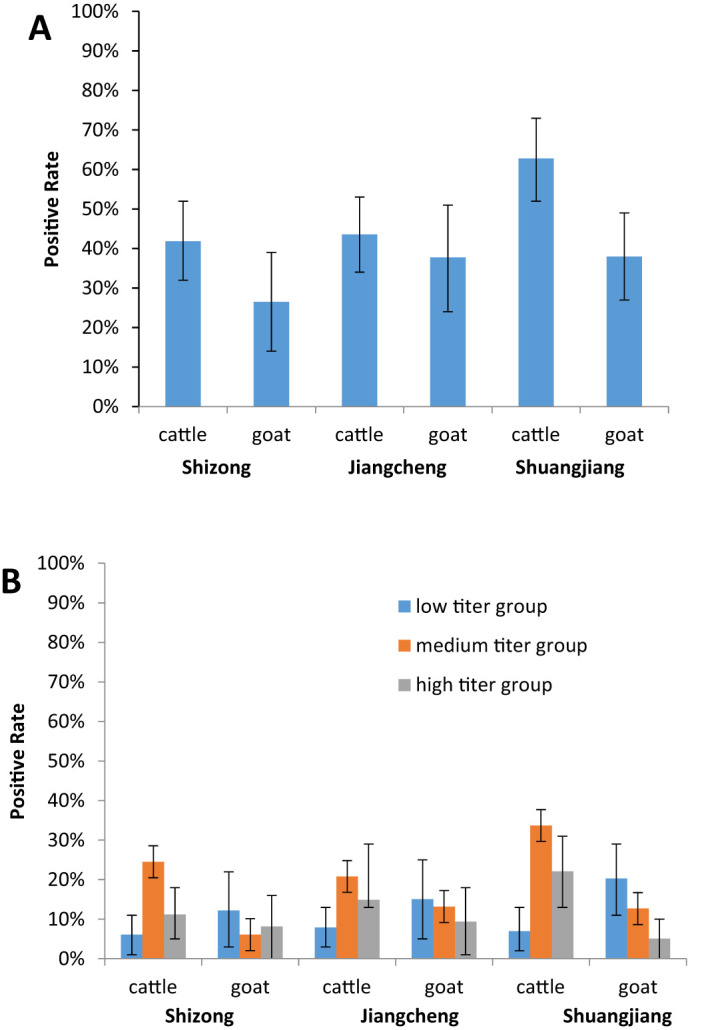
Serology of AKAV in two domestic animals (cattle, and goats) in different cities in Yunnan, China. **(A)** The positive rates of the AKAV antibody in the cattle and goats serum samples between the three sampling sites. **(B)** To evaluate the differences in AKAV neutralizing antibody titers in the cattle and goats serum samples between the three sampling sites.

At the three sampling sites, the positive rate of AKAV neutralizing antibody was higher in cattle serum samples compared to goat serum samples. In Shizong county, the positive rates were 41.84% (41/98, 95% confidence interval (CI): 32–52) for cattle and 26.53% (13/49, 95% CI: 14–39)for goats. In Jiangcheng county, the rates were 43.56% (44/101, 95% CI: 34–53) for cattle and 37.74% (20/53, 95% CI: 24–51) for goats. In Shuangjiang county, the rates were 62.79% (54/86, 95% CI: 52–73) for cattle and 37.94% (30/79, 95% CI: 27–48) for goats (**Figure 3A**).

To assess differences in AKAV neutralizing antibody titers between cattle and goat serum samples across the three sampling sites, the positive serum neutralizing antibody titers were divided into the low titer group (1:4–1:16), medium titer group (1:32–1:128), and high titer group (above 1:256). Most neutralizing antibody titers in cattle serum samples were concentrated in the medium titer group, with positive rates of 58.54% (24/41), 47.73% (21/44), and 53.7% (29/54) at the three sites, respectively. Conversely, the majority of neutralizing antibody titers in goat serum samples were found in the low titer group, with positive rates of 46.15% (6/13), 40% (8/20), and 53.33% (6/30) across the sites (**Figure 3B**).

## 4 Discussion

We isolated two AKAV strains (52 and 55) from biting midges samples collected in Shizong County, Yunnan Province. These strains were shown to infect mammalian kidney cells, resulting in CPE. The complete genome sequences of the two strains revealed presence of three gene segments (S, M, and L). with nucleotide sequences closely resembling those of AKAV. The sequences comparisons indicated highly similar topologies, with only three amino acid variations in the M segment being different. When compared to the whole genome sequences of representative AKAV strains from both domestic and international sources, strains 52 and 55clustered within the same evolutionary branch as AKAV and belonged to the Ia branch of genotype I. The nucleotide and amino acid homology of strains 52 and 55 with other AKAV strains reported in China exceeded 90%. Overall, sequence analysis confirmed, that strains 52 and 55 were indeed AKAV.

In China, seroprevalence studies of AKAV have been conducted in regions such as Guangdong, Sichuan, and Inner Mongolia, with varied results reflecting regional differences in climate, livestock management practices, and vector distribution. For instance, studies from Guangdong reported relatively high seropositivity in cattle populations, likely due to the subtropical climate favoring vector activity year-round (Wang et al., 2019). Conversely, studies in more temperate regions like Inner Mongolia have shown lower AKAV prevalence, correlating with the shorter active season for biting midges vectors (Li et al., 2023). To investigate natural AKAV infection in local domestic animals in Yunnan, three sampling sites were selected to collect 466 serum samples from cattle and goats over one year of age, all without AKAV vaccination records. AKAV antibody detection revealed that 202 samples tested positive, resulting in a positive rate of 43.35%. Notably, the positive rate was higher for cattle (48.77%) than for goats (34.81%), which confirmed that, to a certain extent, cattle were more susceptible to infection than goats under natural conditions (Tang et al., 2019). The three sampling sites, Jiangcheng County, bordering Laos and Vietnam; Shuangjiang County, near Myanmar; and Shizong County, across the river from Guangxi Province. Represent the south, west, and east regions of Yunnan Province, respectively. The serological survey results reflect AKAV prevalence in Yunnan, with moderate to high seroprevalence rates that align more closely with results from subtropical regions like Guangdong, highlighting the influence of climate and ecological factors on AKAV transmission.

AKAV nucleic acid was previously been detected in midges collected from Sichuan and Hainan provinces in China, however, virus isolates were not obtained from these midges (Song et al., 2018; Fan et al., 2020). This study marks the first isolation AKAV from midges in China, with high titer of neutralizing antibodies against the AKAV virus detected in cattle and goat serum samples. These findings confirm that midges are vectors of AKAV, These findings confirm that midges are exists in natural midges in China and forms a natural cycle between midges and local domestic animals in Yunnan Province. The comparison of our findings with previous national and international studies highlights the important role of the midges in AKAV transmission patterns. Importantly, climate change is expected to alter the distribution of biting midges, potentially expanding AKAV’s range into previously unaffected regions. Warmer temperatures and changes in rainfall patterns could lead to longer breeding seasons and increased vector activity in temperate zones, raising concerns about AKAV outbreaks in regions traditionally considered low risk. This has already been observed in Europe, where other vector-borne diseases, such as Bluetongue virus (BTV), transmitted by biting midges, have expanded northward in recent decades due to milder winters and prolonged summers (Mullens et al., 2015; Peter and Estelle, 2015). Given these global changes, the findings from Yunnan have broader implications. Yunnan, with its climate and high livestock density, may serve as a model for understanding how AKAV could spread in other subtropical and temperate regions under changing environmental conditions. Enhanced surveillance and vector control strategies will be essential, not only in Yunnan but also globally, to mitigate the risk of AKAV transmission as climate change continues to reshape vector dynamics.

## Data Availability

The datasets presented in this study can be found in online repositories. The names of the repository/repositories and accession number(s) can be found below: https://www.ncbi.nlm.nih.gov/genbank/, OR387104; https://www.ncbi.nlm.nih.gov/genbank/, OR387105; https://www.ncbi.nlm.nih.gov/genbank/, OR387106; https://www.ncbi.nlm.nih.gov/genbank/, OR387107; https://www.ncbi.nlm.nih.gov/genbank/, OR387108; https://www.ncbi.nlm.nih.gov/genbank/, OR387109.
